# HTLV-2 infection still prevalent among older injecting drug users in Stockholm, Sweden – indications of limited spread to the younger generation

**DOI:** 10.1186/1742-4690-8-S1-A67

**Published:** 2011-06-06

**Authors:** Kerstin Malm, Kristina Hillgren, Sven Britton, Sören Andersson

**Affiliations:** 1 Department of Laboratory Medicine, Örebro University Hospital, Örebro, Sweden; 2 Maria Beroendecentrum, Centre for Dependency Disorders, Stockholm, Sweden; 3 Clinic of Infectious Diseases, Karolinska University Hospital, Solna, Sweden

## Background

High prevalence rates of HTLV-2 have been found among injecting drug users (IDU) in several European countries. In studies in early 1990s among IDUs in Stockholm, HTLV-2 prevalence rates of 2.3% and 3.2 % were found, while HTLV-1 was rare (1-2). We have now, in 2007-8, performed a new study in a similar population in Stockholm, Sweden.

## Objectives

To study if HTLV-2 is still prevalent among IDUs in Stockholm. If so, to see if epidemiological characteristics and risk factors are similar as in 1994.

## Methods

Serum samples from IDUs in Stockholm, during the years 2007-8 (N=1079) were collected and investigated for HTLV-1/2 antibodies. Data regarding age, sex, current drug use, country of origin, detention and homelessness were collected.

## Results

Among 1079 investigated subjects, 35 were found to be positive for antibodies to HTLV-1 and/or HTLV-2, giving an overall HTLV-prevalence of 3.2%. Of these, 2 (0.2%) had antibodies to HTLV-1, 28 to HTLV-2 (2.6%) and 5 (0.5%) had non-typeable antibodies to HTLV. The overall study group had similar age and sex distribution as in 1995. However, the HTLV-positive individuals were 10 years older than the HTLV-negative group (mean age) and compared to the cases studied in 1995.

## Conclusion

The HTLV prevalence among IVDUs in Stockholm, Sweden, 2007-8 was comparable to the rates found in the early 1990s. As back then, the HTLV-positive IVDUs were significantly older than the HTLV-negative persons. However, in the present study the age difference was more pronounced, 10 years vs 4 years mean difference. The higher age of HTLV cases indicates either a spread of HTLV mainly among older individuals, little spread to the younger persons or possibly limited spread of HTLV in general, since the HTLV-infected cases are now more than 10 years older than in 1995.

